# The Role of Parathyroid Hormone Level as a Predictor of Hypocalcemia After Total Thyroidectomy for Thyroid Cancer: A Cross-Sectional Study

**DOI:** 10.7759/cureus.78897

**Published:** 2025-02-12

**Authors:** Fernando Semanate, Wilmer Tarupi, Tatiana Fernandez Trokhimtchouk, Christian Palacios, Oscar Jaramillo

**Affiliations:** 1 Surgical Oncology, Hospital de Especialidades Carlos Andrade Marin, Quito, ECU; 2 National Tumor Registry Coordination, Hospital Oncológico Solón Espinosa Ayala (SOLCA), Quito, ECU; 3 General Surgery, Universidad Internacional del Ecuador/Axxis Hospital, Quito, ECU; 4 Head and Neck Surgery, Hospital de Especialidades Carlos Andrade Marin, Quito, ECU

**Keywords:** low calcium, open thyroidectomy, parathyroid level, postoperative parathyroid hormone (pth), thyroid cancer surgery, thyroidectomy complications

## Abstract

This study aimed to investigate the utility of measuring parathyroid hormone (PTH) levels as a predictor of hypocalcemia in a population of patients undergoing total thyroidectomy for thyroid cancer between 2016 and 2019. We conducted an observational, analytical, descriptive, cross-sectional investigation, assessing PTH levels as a predictor of hypocalcemia following thyroidectomy. Among patients with hypoparathyroidism, 25.5% experienced hypocalcemia, while 74.5% had normal serum calcium levels. The likelihood of hypocalcemia was five times higher in patients with hypoparathyroidism (OR: 5.43; 95% CI: 1.89-15.6), a statistically significant finding (p < 0.05). Additionally, PTH values at 24 hours post-surgery averaged 28.9 pg/mL (SD: 30.8 pg/mL), ranging from 0.01 to 235 pg/mL. Serum calcium levels averaged 8.31 mg/dL (SD: 0.74), with values ranging from 6.5 to 10.6 mg/dL. The study demonstrates a statistically significant association between PTH levels and post-surgical serum calcium levels, albeit with moderate predictive power. These findings support the utility of PTH measurement in predicting hypocalcemia following thyroidectomy, underscoring its potential clinical relevance in patient management.

## Introduction

A marked increase in the incidence of thyroid cancer has been evident worldwide over the past three decades. In Ecuador, Quito has reported some of the highest incidence rates globally, with 10.1 cases per 100,000 men and 47.0 cases per 100,000 women [1], placing it among the areas with the highest indicators globally [2].

Total thyroidectomy is the primary treatment for thyroid cancer, with postoperative hypocalcemia being one of its most common complications, occurring in up to 50% of cases. This complication results from direct trauma, devascularization, or unintentional removal of the parathyroid glands during surgery, leading to transient or permanent hypoparathyroidism [3].

The diagnosis of hypocalcemia is made with serum calcium values associated with clinical symptoms in up to 30% of cases. Typically, this pathological condition presents acutely after hospital discharge in emergency departments. Consequently, strategies have been developed for early, even subclinical, diagnosis, including measurement of total calcium, ionized calcium, and parathyroid hormone (PTH) levels [4].

Serum levels of PTH are regulated by a negative feedback loop. PTH is secreted by the parathyroid glands as serum calcium levels decrease, prompting bones to release more ionized calcium and stimulating the kidney and intestines to reabsorb it [5]. The release of PTH is reduced as serum calcium levels increase. The literature suggests that measuring PTH in the immediate postoperative period can predict hypocalcemia and guide calcium supplementation, potentially reducing hospital stays and complications [6,7].

The aim of this study is to analyze PTH levels as predictors of hypocalcemia in patients undergoing total thyroidectomy for thyroid cancer at a tertiary hospital in Ecuador, within a cohort studied from 2016 to 2019. Unlike previous studies that focused on early postoperative PTH measurements (two, four, or six hours), our study evaluates the utility of PTH levels at 24 hours post-surgery, as a predictor of hypocalcemia, which aligns with institutional protocols and allows for a more practical decision-making approach in resource-limited settings [8].

## Materials and methods

Study design and population

We conducted a cross-sectional study to evaluate PTH levels as a predictor of hypocalcemia in patients undergoing total thyroidectomy for thyroid cancer at a tertiary hospital in Ecuador. The study spanned from 01 September 2016 to 31 July 2019. This time frame refers to data collection rather than patient follow-up, making a cross-sectional design more appropriate than a retrospective cohort study.

Inclusion and exclusion criteria

Patients diagnosed with thyroid cancer undergoing total thyroidectomy were included in the study. Eligible participants were adults aged 18-80 years, of both sexes. To ensure a representative sample, age groups were categorized as young adults (18-40 years), middle-aged (41-60 years), and elderly (61-80 years). Patients with preoperative hypocalcemia or hypoparathyroidism, individuals with chronic kidney disease or other metabolic disorders affecting calcium homeostasis, and cases with incomplete medical records were excluded from the study. The selection criteria ensured that the study population was homogeneous and that factors influencing calcium metabolism were controlled.

Sample size

A total of 148 patients met the predefined selection criteria and were included in the study.

Ethical approval

This study was approved by the Institutional Ethics Committee (IEC) of Sociedad de Lucha contra el Cáncer (SOLCA), Núcleo de Quito under approval number MSP-VGVS-2021-0193-0, approved on 21 May 2021, Additionally, approval was reaffirmed by Oficio N°073-2022 CEISH Quito, issued on 21 September 2022. Ethical approval was obtained retrospectively for the analysis of pre-existing medical records, following institutional and international guidelines for cross-sectional studies.

Data collection and statistical analysis

Descriptive statistics were presented as frequencies and percentages. Univariate analysis included measures of central tendency and dispersion for quantitative variables. Bivariate analysis was performed using the Chi-square test (χ²), with statistical significance set at p < 0.05. Receiver Operating Characteristic (ROC) curve analysis was employed to determine the diagnostic accuracy of PTH for predicting hypocalcemia. Statistical analysis was conducted using IBM SPSS Statistics for Windows, Version 23 (Released 2015; IBM Corp., Armonk, NY, USA). The ROC curve was used to evaluate the predictive value of PTH levels in identifying postoperative hypocalcemia risk.

## Results

During the study period from 2016 to 2019, a total of 32,501 patients underwent thyroidectomy. Among them, 148 patients met the predefined selection criteria and were included in the study (Table 1).

**Table 1:** Demographic characteristics of the population

Age	Frequency (n)	Percentage (%)
Young adult (18-29 years)	5	3.4
Middle-aged adult (30-59 years)	97	65.5
Elderly adult (≥60 years)	46	31.1
Sex		
Male	23	15.5
Female	125	84.5

In the fine-needle aspiration biopsy (FNAB) results, classified according to the Bethesda system, category V (Suspicious for Malignancy; n = 84, or 56.8%) and category VI (Malignant; n = 45, or 30.4%) were predominant. Total thyroidectomy was the standard surgical treatment for all patients, with central neck dissection performed in 43.9% of cases. Histopathological examination revealed papillary thyroid carcinoma in 64.9% of cases and follicular carcinoma in 35.1%.

Postoperatively, the mean PTH level at 24 hours was 28.9 pg/mL (SD: 30.8 pg/mL), while the mean serum calcium level was 8.31 mg/dL (SD: 0.74). Using a cutoff value of 10 pg/mL for PTH, hypoparathyroidism was diagnosed in 47 patients (31.8%). Hypocalcemia, defined by a serum calcium level below 7.5 mg/dL, was identified in 18 patients (12.2%). Clinical symptoms of hypocalcemia were evident in 12 patients (8.1%), all of whom received oral calcium and calcitriol.

PTH as a predictor of postoperative hypocalcemia

Table 2 shows that, among patients with hypoparathyroidism, 25.5% experienced hypocalcemia, while 74.5% had normal serum calcium levels. The likelihood of developing hypocalcemia was found to be five times higher in patients with hypoparathyroidism (OR: 5.43; 95% CI: 1.89-15.6), reaching statistical significance (p < 0.05).

**Table 2:** Parathyroid hormone as predictor of hypocalcemia in patients post-total thyroidectomy Statistical test used: Chi-square test (χ²); p < 0.05 considered significant

Parathyroid hormone	Postoperative serum calcium level (mg/dL)	OR (95% CI)	Chi-square (χ²)	p-value
Low (<10 pg/mL)	Hypocalcemia (<7.5) (n = 12, or 25.5%)	5.43 (1.89-15.6)	10.52	0.001
	Normocalcemia (≥7.5) (n = 35, or 74.5%)			
Normal (≥10 pg/mL)	Hypocalcemia (n = 6, or 5.9%)			
	Normocalcemia (n = 95, or 94.1%)			

Building upon the data from Table 2, we computed parameters to evaluate the efficacy of PTH in predicting immediate postoperative hypocalcemia. Our analysis revealed a sensitivity of 66.7% (95% CI: 41.2-85.6) for this parameter. However, this value falls short of the threshold required for diagnostic testing, which necessitates a sensitivity of 95% or higher.

In terms of specificity, it was determined to be 73.1% (95% CI: 64.5-80.3). This parameter assesses the test's accuracy in correctly identifying patients without the condition. While the specificity value falls short of the ideal, it surpasses the sensitivity, suggesting that PTH determination may be somewhat more effective in ruling out postoperative hypocalcemia.

The positive predictive value (PPV), indicating the likelihood of hypocalcemia in patients with hypoparathyroidism, stood at 25.5% (95% CI: 14.4-40.6). This reinforces the earlier observation regarding sensitivity, particularly in cases where PTH levels are below 10 pg/mL.

Conversely, the negative predictive value (NPV), reflecting the probability of hypocalcemia absence in patients without hypoparathyroidism, reached 94.6% (95% CI: 87.0-97.6). This underscores the test's utility in excluding hypocalcemia.

The positive likelihood ratio (LR+) was calculated at 2.48 (95% CI: 1.61-3.82), while the negative likelihood ratio (LR-) was 0.46 (95% CI: 0.24-0.88). These values indicate the likelihood of hypoparathyroidism in patients with hypocalcemia (LR+) and the likelihood of normal PTH levels in patients without hypocalcemia. Overall, these results suggest that the test's performance in predicting post-total thyroidectomy hypocalcemia is modest (Table 3).

**Table 3:** PTH level for prediction of postoperative hypocalcemia PTH: parathyroid hormone

Parameter	%	CI 95%
Sensitivity	66.7	41.2-85.6
Specificity	73.1	64.5-80.3
Positive predictive value	25.5	14.4-40.6
Negative predictive value	94.6	87.0-97.6
Positive likelihood ratio	2.48	1.61-3.82
Negative likelihood ratio	0.46	0.24-0.88
Diagnostic odds ratio	5.43	1.89-15.6

**Figure 1:**
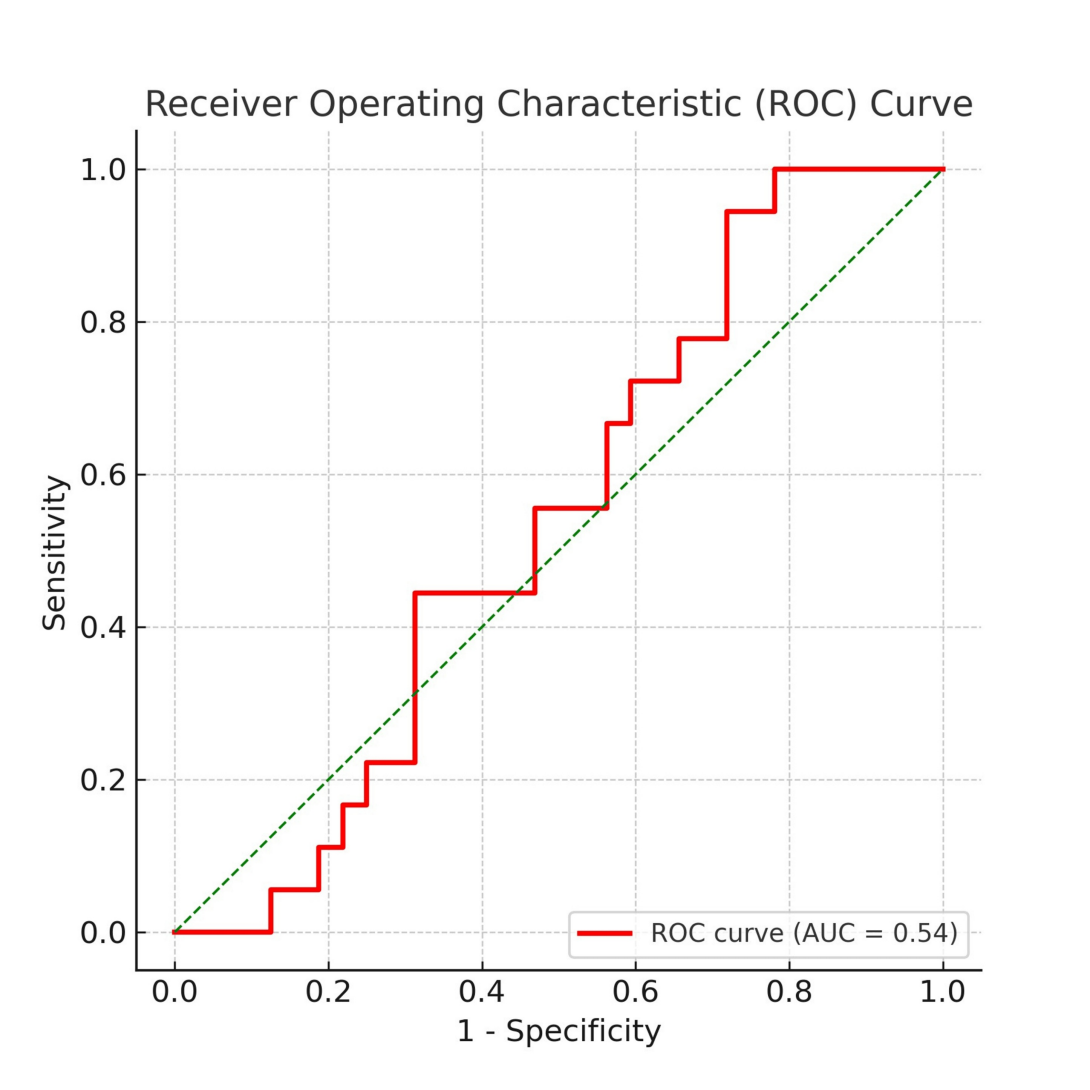
Receiver operating characteristic (ROC) curve

To further assess the predictive value of PTH levels in detecting postoperative hypocalcemia, we performed an ROC curve analysis (Figure 1). The ROC curve illustrates the relationship between sensitivity and 1-specificity, with an area under the curve (AUC) of 0.54, indicating a modest predictive ability of PTH levels for hypocalcemia. While a higher AUC value would suggest stronger discrimination between hypocalcemic and normocalcemic patients, the observed value suggests that PTH alone may not be a robust predictor and should be interpreted in conjunction with other clinical parameters. The ROC curve also highlights the trade-off between sensitivity and specificity at various cutoff points, reinforcing the need for further refinement in predictive modeling.

The present study also involved the development of a linear regression model to explore the relationship between PTH values and serum calcium levels 24 hours post-surgery. The model yielded an adjusted coefficient of correlation (adjusted R² = 0.054), indicating that only 5.4% of the variability in serum calcium levels at 24 hours can be attributed to fluctuations in PTH (standard error = 0.72).

Furthermore, Table 4 provides evidence of a statistically significant association between these variables (p = 0.03).

**Table 4:** Summary of ANOVA (simple linear regression) Dependent variable: total calcium (mg/dL); Predictors: (constant), PTH value 24 hours post-procedure; "b" indicates statistical significance in the regression model (p = 0.03) ANOVA: analysis of variance; PTH: parathyroid hormone

Model	Sum of squares	df	Mean square	F	Sig.
Regression	4.880	1	4.880	9.436	0.003^b^
Residual	75.513	146	0.517	-	-
Total	80.393	147	-	-	-

## Discussion

The risk of developing hypocalcemia was found to be five times higher among those with hypoparathyroidism compared to those without it (OR: 5.43; 95% CI: 1.89-15.6, p < 0.05). Lončar et al. [9] detected a higher frequency of post-surgery hypocalcemia in patients with low PTH levels, with 15.1% of all included patients experiencing this complication. Similarly, other authors identified a higher incidence of hypocalcemia; out of 310 patients studied, 34% (n = 108) experienced hypocalcemia and required immediate oral calcium treatment post-surgery [10].

PTH has been described in the medical literature as a paraclinical study serving as a predictor of hypocalcemia after thyroidectomies. Hence, its effectiveness as a predictor was determined, reaching moderate sensitivity at 66.7%. However, other researchers, such as Del Rio et al. [11], found 100% sensitivity for a decrease of over 80% in PTH levels during the immediate postoperative period, necessitating early and effective treatment with a 98% reduction. Izquierdo et al. [12] defined optimal PTH cutoff values for predicting hypocalcemia, recommending its use despite its high economic cost. This recommendation was supported by Gutiérrez Fernández et al. [10], who reported 100% sensitivity, without false negatives, for various PTH cutoff values.

Regarding specificity, the reduction in PTH values reached a level of 87% in predicting hypocalcemia, while a reduction of up to 98% showed 98.6% specificity in determining the need for exogenous calcium supplementation. However, our ROC curve analysis revealed that the predictive utility of PTH for postoperative hypocalcemia was "moderate" (AUC = 64.5%; 95% CI: 48.4-80.5). Conversely, Triguero Cabrera et al. [13] found a "high" predictive power of PTH reduction for hypocalcemia.

While previous studies have assessed PTH at two, four, or six hours postoperatively, our study evaluates 24-hour PTH levels as a predictor of hypocalcemia. Literature indicates that a decrease in PTH levels within the first 24 hours, particularly among asymptomatic women, correlates with a sensitivity exceeding 61% in predicting hypocalcemia [14]. Additionally, researchers have found that a reduction of up to 80% in postoperative PTH values can achieve 100% sensitivity for predicting hypocalcemia, supporting the notion that later PTH measurement may enhance diagnostic accuracy [15]. In our institutional setting, PTH testing at 24 hours is the standard protocol, facilitating practical decision-making regarding calcium supplementation.

We conducted a linear regression model to determine the relationship between detected PTH values and serum calcium at 24 hours post-surgery, revealing that only 5.4% of the variance in calcium levels could be attributed to PTH reduction (adjusted R² = 0.054). Nevertheless, there was a statistically significant association between PTH and serum calcium levels in the immediate postoperative period, as demonstrated by analysis of variance (ANOVA) (p = 0.03) and linear regression analysis. These findings were consistent with those of Filho et al. [16], who observed a relationship between low PTH levels and post-thyroidectomy hypocalcemia.

In summary, although PTH shows promise as a predictor of post-thyroidectomy hypocalcemia, its diagnostic accuracy may vary depending on the study population and methodology, additional research is needed to elucidate the optimal cutoff values and timing of PTH measurements for predicting hypocalcemia accurately. Moreover, the inclusion of other factors, such as preoperative vitamin D levels, in future studies may further enhance predictive models for post-thyroidectomy hypocalcemia.

The study has several limitations. The small sample size limits the generalizability of the findings. Since this was a single-center study, the results may not reflect broader population trends. The high cost of PTH testing restricts accessibility in low-resource settings, which could impact the feasibility of routine implementation. Additionally, confounding factors, such as preoperative vitamin D levels, were not analyzed, which might have influenced calcium metabolism and the study outcomes.

## Conclusions

Our study demonstrates a statistically significant correlation between PTH levels and postoperative serum calcium levels, albeit with "moderate" predictive power. We found that PTH levels at 24 hours post-surgery averaged 28.9 pg/mL (SD: 30.8 pg/mL), ranging from 0.01 to 235 pg/mL. Similarly, serum calcium levels averaged 8.31 mg/dL (SD: 0.74), with values ranging from 6.5 to 10.6 mg/dL. These findings reinforce the importance of monitoring for hypocalcemia in this patient population, given its frequency.
